# Intra-Amoeba Multiplication Induces Chemotaxis and Biofilm Colonization and Formation for *Legionella*

**DOI:** 10.1371/journal.pone.0077875

**Published:** 2013-10-24

**Authors:** Renaud Bigot, Joanne Bertaux, Jacques Frere, Jean-Marc Berjeaud

**Affiliations:** 1 Equipe Microbiologie de l’Eau, Ecologie & Biologie des Interactions, Centre national de la recherche scientifique UMR 7267, Université de Poitiers, Poitiers, France; 2 Equipe Ecologie Evolution Symbiose, Ecologie and Biologie des Interactions, Centre national de la recherche scientifique UMR 7267, Université de Poitiers, Poitiers, France; University of Louisville, United States of America

## Abstract

*Legionella pneumophila*, a facultative intracellular bacterium, is the causative agent of legionellosis. In the environment this pathogenic bacterium colonizes the biofilms as well as amoebae, which provide a rich environment for the replication of *Legionella*. When seeded on pre-formed biofilms, *L. pneumophila* was able to establish and survive and was only found at the surface of the biofilms. Different phenotypes were observed when the *L. pneumophila*, used to implement pre-formed biofilms or to form mono-species biofilms, were cultivated in a laboratory culture broth or had grown intracellulary within the amoeba. Indeed, the bacteria, which developed within the amoeba, formed clusters when deposited on a solid surface. Moreover, our results demonstrate that multiplication inside the amoeba increased the capacity of *L. pneumophila* to produce polysaccharides and therefore enhanced its capacity to establish biofilms. Finally, it was shown that the clusters formed by *L. pneumophila* were probably related to the secretion of a chemotaxis molecular agent.

## Introduction

*Legionella* is a facultative intracellular gram-negative bacteria found in natural freshwater environments as well as in man-made water systems [1]. *Legionella pneumophila* is the major agent of legionellosis, except in Australia, where *Legionella longbeachae* is the major causative agent [2], [3]. The infection is due to multiplication within alveolar macrophages after inhalation by human of contaminated aerosols produced by many engineered water systems such as air conditioning, cooling towers, hot water systems, whirlpools and spas, vegetable misters, shower heads and dental unit water lines [4]–[8].

The control of *L. pneumophil*a in water networks is principally based on decontamination processes. However, it is now well known that this bacterium resists to biocide treatments because of its sessile way of life into biofilms as well as its capacity of growth within amoeba [9]–[11].

*Legionella spp*. require supplementation in amino acids and therefore, have developed mechanisms to acquire these nutrients by residing in nutrient rich biofilms [12]–[15]. *L. pneumophila* is able to survive and grow on dead biofilm-associated microbial cells such as heat-killed bacteria, amoebae and yeast [16]. In *Legionella* monospecies biofilm development in rich nutrient medium, temperature has a role about thickness and structure [17]. Biofilms constitute the major source of human contamination. Microbial formation of biofilm communities provides protection against physical or chemical agents. Thus, it was demonstrated by numerous studies [13], [14], [18] that *L. pneumophila*, prepared from laboratory culture medium, is able to implement into a pre-formed biofilm for bacteria.

Amoeba such as *Acanthamoeba castellanii* and ciliate protozoan are able to graze biofilm material [19]. In these environments, *Legionella* is a target of protozoan predation and has developed the capacity to parasitize and reside in 20 species of amoebae, three species of ciliated protozoa and one species of slime mould [20]–[22]. Numerous studies show the necessity of the presence of protozoans for the survival and replication of *L. pneumophila* in aquatic environments [14], [23]. The study of Declerck et al, in 2007 [24], shows that *Acanthamoeba castellanii* is not only used for intracellular replication but as a transporter like a “Trojan horse” [12] and Neumeister et al [25] proved that intracellular replication in human monocytic leukocytes is enhanced after infection of *Acanthamoeba castellanii*. Free protozoa not only select virulence traits, but also facilitate the transmission of *Legionella* spp to humans within intact amoebae or expelled vesicles [26]. The ability of *Legionella* spp to persist and colonize biofilm as well as parasitization of amoeba cells can be considered as a survival mechanism, but this can lead to severe consequences for human health.

With the current study, we provide information about the implementation of *Legionella* on static biofilms composed of non-*Legionella* bacteria similar to those present in aquatic systems. We compared the behavior of *Legionella* after culture in a usual medium, after infection of amoebae, or passage through ciliated protozoan in condition near to anthropogenic water networks.

## Experimental Procedures

### Strains and culture conditions

The *Legionella* strains used are *L. pneumophila* Lens (CIP 108286), a clinical isolate, Paris (LG03), an environmental isolate and *Legionella longbeachae* (ATCC 33484). The plasmid PSW001 or plasmid pMMB207-KM14-GFPc [26] encoding the protein DsRed and GFPMut2 were used to transform the bacteria. *Legionella* was grown in BYE medium (Buffered Yeast Extract) or BCYE (Buffered Charcoal Yeast Extract) at 37°C with chloramphenicol at 10 µg/mL when necessary. The experiments were made with *L. pneumophila* expressing GFP or Ds-red with similar results.

The pre-formed biofilms consisted of selected strains usually found in anthropogenic water networks [22]. Non-Legionella bacteria were *Aeromonas hydrophila* (Library of Microbiology Universiteit Gent, Belgium (LMG 2844)), *Escherichia coli* (LMG 2092), *Flavobacterium breve* (LMG 4011) and *Pseudomonas aeruginosa* (LMG 1242). These strains were grown in BHI (Brain Heart Infusion) at 37°C.

*Acanthamoeba castellani* 30254 [27] was grown in medium PYG (Proteose Yeast Glucose) [28] at 25°C and *Tetrahymena tropicalis* was cultivated in PCB (Plate count Broth) at room temperature in the dark.

### Preparation for the implementation of *Legionella* in the biofilm

The preparation described downhere were realized with the three *Legionella* strains cited previously.

#### “Medium Grown” (MG) *Legionella* non-filamentous condition

*Legionella* grown in culture media produced numerous filamentous cells, often more than 40 µm long, which is not the case after their passage into amoebae or ciliates (data not shown). *Legionella* non-filamentous stationary phase form were grown according to the protocol of the CNRL Lyon [10]. Bacteria were then suspended in sterile mineral water (Evian). As it is well known that nutrient depletion induces the stationary phase in *Legionella* [29], [30], under these conditions, these bacteria reached stationary form and were named "Medium Grown” bacteria. The fluorescence of strains was checked by epifluorescence microscopy.

#### "Amoeba Grown" (AG) *Legionella*

Amoeba, after 2 days of culture, were harvested and washed in amoeba buffer. *Acanthamoeba* were placed in contact with *Legionella* following a multiplicity of infection or MOI (Mutiplicity Of Infection) of 0.1 and incubated at 30 ° C. After 96 hours, *Legionella* were collected by centrifugation at 10000 g for 10 minutes. The pellet was washed twice with filtered mineral water and was resuspended by vortex. After centrifugation, the preparation was allowed 10 min to precipitate and only the upper half of the volume was taken. The absence of amoeba and the fluorescence of *Legionella* were checked using an epifluorescence microscope. Under these conditions, these bacteria were designated “Amoeba Growing" (AG).

#### “Medium Growing” *Legionella* treated with AG supernatant (MG+ Sur AG)

After 24 hours of incubation at 37°C, AG *Legionella* suspension was centrifuged 5 minutes at 10000 g and the cell free supernatant was collected. Supernatant (1 mL) was added to a pellet of 1.10^8^ MG cells. After incubation during 1 hour at 37°C, *Legionella* suspension was centrifuged at 4500 g for 15 minutes and washed two times with mineral water. The fluorescence of *Legionella* was checked using an epifluorescence microscope. Under these conditions, these bacteria were designated “treated MG *Legionella*" (tMG).

#### “Ciliated Grown” (CG) *Legionella*

After 7 days of culture *of Tetrahymena tropicalis* the medium was gradually replaced between centrifugation by Osterhout's Tris buffer [10]. *Legionella* were added with MOI of 1000 bacteria for 1 protozoan. After 96 hours, the suspension was centrifuged and the medium discarded. Distilled water was added and left one night in the dark. The next day, water was eliminated. *Legionella* pellets were broken using syringes 27 and 22 gauges. Broken pellets and *Legionella* fluorescence were controlled by epifluorescence microscopy. Under these conditions, these bacteria and were designated “Ciliate Grown" (CG).

### Alcian blue test

The wells of 6 well plates were filled with 2 ml of sterile mineral water. Each well was inoculated with 2.10^9^ *Legionella* and incubated 24 hours at 37°C without agitation. The supernatant was removed and replaced by 1 mL of Alcian blue 8G (Sigma-Aldrich) at 0.1% in distilled water. After 30 min incubation, each well was washed three times and 1 mL of 33% acid acetic was added. Absorbance was measured at 595 nm.

### Crystal violet test

The first part of the test was performed like Alcian blue assay. The supernatant of the wells was removed and replaced by 1 mL of 0.3% Crystal Violet (Sigma-Aldrich) in distilled water. After 15 min incubation, each well was washed three times with mineral water and 1 mL of absolute ethanol was added. Absorbance was then measured at 595 nm.

### *Legionella* transformation

*Legionella* were grown on BCYE for 72 h and suspended by scrapping from the plates with 50 mL of sterile double distilled water. The cell suspension was centrifuged in a cold rotor (4°C) at 5500 g for 10 min. The supernatant was discarded and the pellet was washed three other times with half a volume of water each time. The final volume was added to make a concentration of 2.10^11^ cells/mL. The bacteria suspension was dispensed into 50 µL and transferred to a cold electroporation cuvette. After addition of 200 µg of plasmid DNA, electroporation was performed using a Gene Pulser apparatus. One mL of BYE was added after the pulse and incubated at 37°C without agitation. After one hour, 100 µL of suspension were spread on BCYE plates containing chloramphenicol. Transformants were confirmed by the presence of plasmid DNA by electrophoresis on 0.4% agarose gels.

### Microscopy

#### Development of biofilms

Biofilms were made in 12 well plates with glass bottom adapted for confocal microscopy. In each well, 2 mL of BHI diluted 1/100 with mineral water (to reach organic matter concentration equivalent to river water) were introduced. The wells were inoculated with 5.10^6^ bacteria of each of the four non-*Legionella* strains from an overnight pre-culture. The plates were incubated for 15 days at 37°C with media changes every four days. The medium was then removed and replaced by 2 mL of filtered mineral water. Biofilms were then seeded with 1.10^7^ *Legionella* cells of different types (MG, AG and CG) and the plates were incubated for 7 days at 37°C. The microscopic observations were realized every 24 hours.

#### Staining

The wells were washed with mineral water to eliminate non-adherent *Legionella*. To mark the exopolysaccharide matrix, Concanavalin A AlexaFluor 350 conjugate (Invitrogen) was added according to the manufacturer's recommendations and wells were incubated 20 minutes at 37°C and then washed with mineral water. Draq 5 (Biostatus) was added to stain all bacteria as recommended by the supplier.

#### Observation and processing

The observations were made with the epifluorescence microscope Axio Observer A1 (Zeiss) equipped with Apotome. This module allows structured illumination. The microscope is equipped with a mercury lamp and filters specific for DAPI (Zeiss Filter Set 49), FITC (Zeiss Filter Set 44), Cy3 (Zeiss Filter Set 43) and Cy5 (Zeiss Filter Set 50), and the Plan Neofluar 1.25 oil immersion objective (Zeiss). The images were taken using the AxioVision software and processed with the software Imasis7.4.1 and Zen (Zeiss).

#### PCR

DNA extractions were performed with the High Pure PCR Template Kit (Roche) according to the supplier's recommendations. Primers used were listed in Table 1. PCR conditions were as follows: an initial denaturation step of 94°C for 5 min and then 30 cycles of 94°C for 30 sec., primer annealing 60°C for 30 sec., and polymerase extension at 72°C for 60 sec.

**Table 1:** Sequence and name of primers used for tested presence or absence of *lqs* gene.

Name	Sequence
*lqsA* forward	AATCCTGGCAAGGGAAACAC
*lqsA* reverse	AACACGGCTCCAAAGATGTC
*lqsR* forward	CGTATTGCAGCTGGATGAGG
*lqsR* reverse	GGCAGGAACCAAACAATTCG
*lqsS* forward	CTGGTTGCCTCAGCCTTATG
*lqsS* reverse	AGCGTATTCGCCCTCTTTAG
*lqsT* forward	GATGCAAGCCACCAAATCAC
*lqsT* reverse	GCAAGCGGCGTTCTTAAATC

#### Quantitative PCR

DNA extractions were performed with the High Pure PCR Template Kit (Roche) according to the supplier's recommendations. The qPCR were performed with primers specific for the *mip* gene (mipA1, mipA2 described by Jonas et al [31], with the Light Cycler FastStart DNA kit SybrGreen Master and Light Cycler 32 wells according to the manufacturer's recommendations.

## Results

### Implementation of *Legionella* on multi-species biofilms

It was shown earlier [32] that *Legionella pneumophila* is able to colonize pre-formed biofilms consisting of the four aquatic bacteria *Aeromonas hydrophila*, *Escherichia coli*, *Flavobacterium breve* and *Pseudomonas aeruginosa*, generally present in the same aquatic environment as *L. pneumophila* [12], [14], [18], [32]–[34]. Thus we prepared biofilms composed of these bacteria, on glass support fixed under 12 wells microplates and using a BHI medium diluted 100 times with Evian mineral water. Indeed, it was recently shown [35] that the mineral composition of this water is particularly adapted for the biofilm formation by *L. pneumophila*. Every 4 days, medium was renewed. In all conditions tested, *Legionella* were described as highly mobile [36], [37].

**Figure 1:**
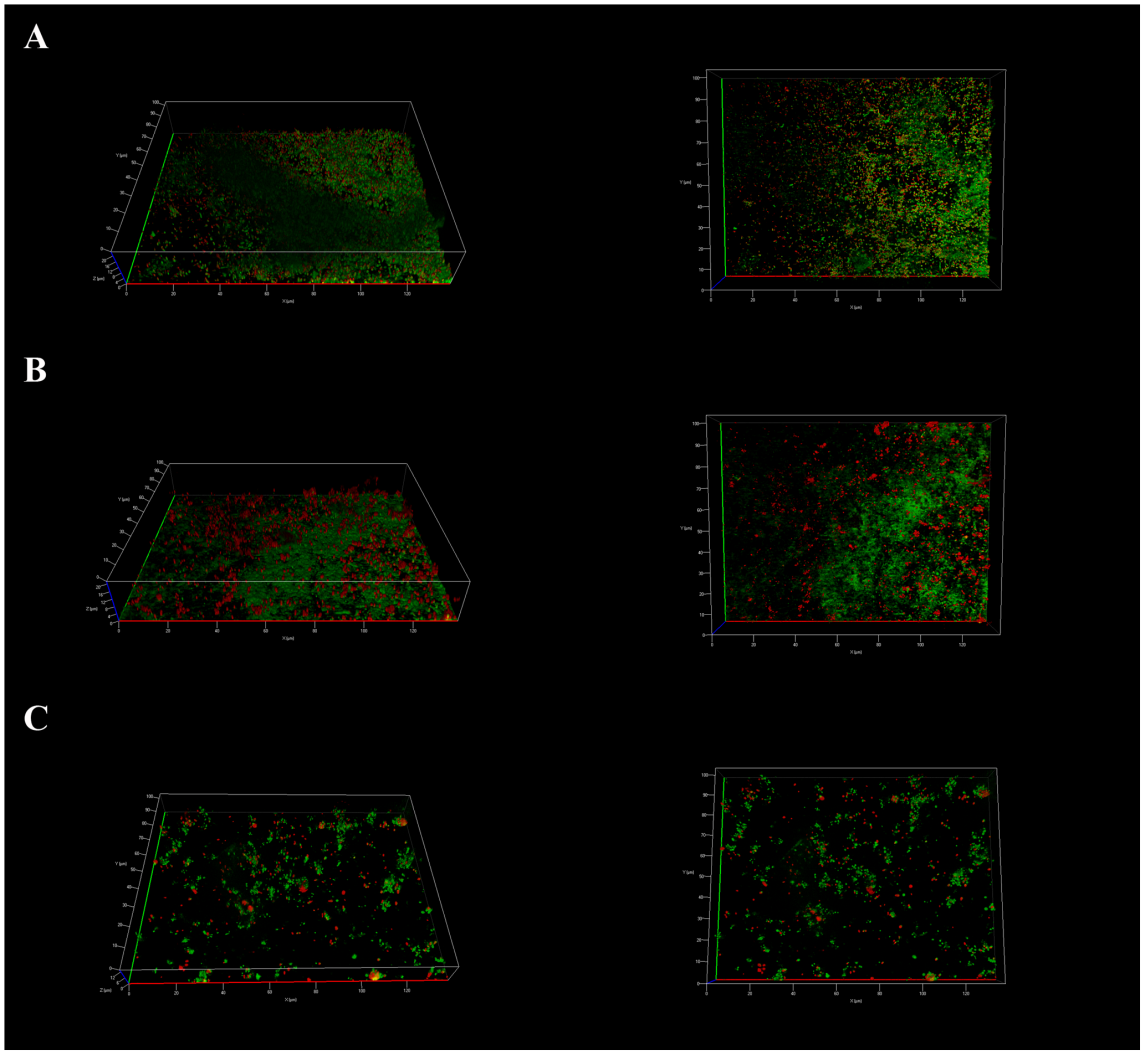
Apotome imaging, 7 days after establishment of *L. pneumophila* Lens A) MG, B) AG and C) CG, in biofilm pre-formed with four non-Legionella strains during 15 days from water networks. *L. pneumophila* expressing Ds-Red marker appears in red and non-*Legionella* bacteria, marked using Draq5 stain, in green. Pictures are representative of 3 independent experiments.

**Figure 2:**
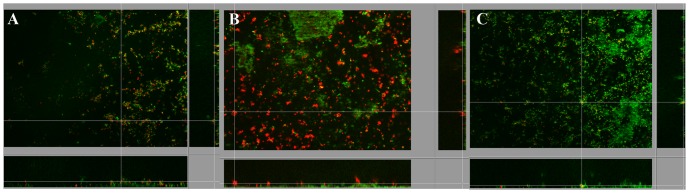
Orthogonal views of apotome imaging, 7 days after establishment of *L. pneumophila* Lens A) MG and B) AG, in biofilm pre-formed with four non-Legionella strains during 15 days from water networks. *L. pneumophila* expressing Ds-Red marker appears in red and non-*Legionella* bacteria, marked using Draq5 stain, in green.

After 15 days, Ds-red fluorescent *L. pneumophila* Lens cells, prepared from BYE culture medium, named “Medium Grown” (MG) or after growing within *Acathamoeba castelanii*, “Amoeba Grown” (AG) or within ciliated protozoan, *Tetrahymena tropicalis*, "Ciliated Grown" (CG) were added to the pre-formed biofilms and medium was replaced by steril mineral water (Evian). Within one week, MG *L. pneumophila* established in the pre-formed biofilms as single cells, which were globally disseminated overall the surface of the non-*Legionella* strains biofilm (figure 1A). Transversal imaging (Figure 2) demonstrated that none of the *L. pneumophila* cells were found inside the biofilm structure but were all situated at the surface of the biofilm. The same results were obtained with CG *L. pneumophila,* (Figure 1C). Likewise, AG *L. pneumophila* were found only on the surface of the biofilm (Figure 1B) and were never found deeper inside the structure. However, contrarily to the MG bacteria the AG *L. pneumophila* appeared aggregated instead of individual cells. The same results were obtained with *L. pneumophila* Paris and *L. longbeachae* (not shown).

**Figure 3:**
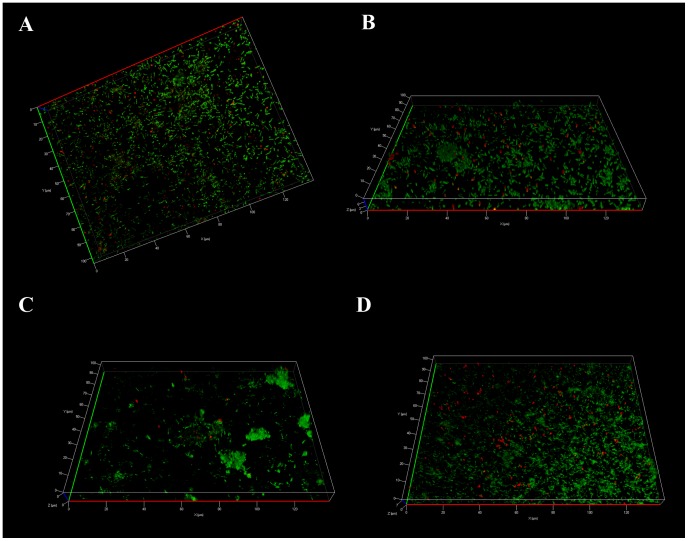
Apotome imaging, 7 days after establishment of *L. pneumophila* Lens MG, in biofilm pre-formed with each of the four non-Legionella strains during 15 days from water networks. Establishment in A) *Pseudomonas aeuginosa*, B) *Flavobacterium breve*, C) *Aeromonas hydrophila* and D) *Escherichia coli*. *L. pneumophila* expressing Ds-Red marker appears in red and non-*Legionella* bacteria, marked using Draq5 stain, in green.

When inoculated to monospecies biofilms pre-formed with each of the four non-*Legionella* strains, the MG and AG *L. pneumophila* had the same behavior as observed with multispecies biofilms, namely single cells for MG *Legionella* and clusters for AG *Legionella* (Figure 3). These results indicate that none of the four strains constituting the pre-formed biofilm could be specifically implicated in the establishment differences observed for the types of *L. pneumophila*.

### Monospecies biofilm formation by *L. pneumophila*

**Figure 4:**
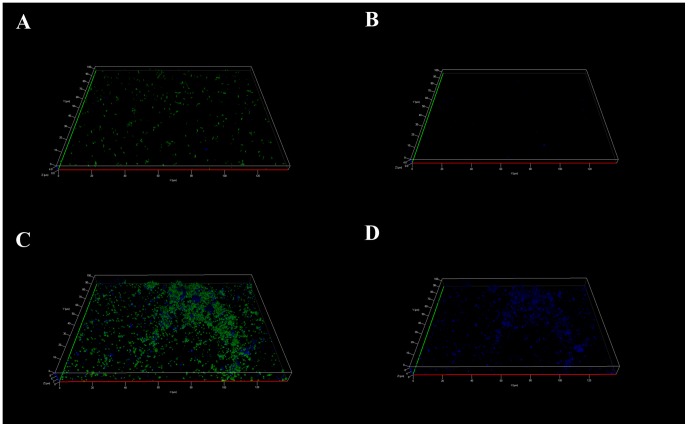
Apotome imaging after 7 days of biofilm development on a glass surface of *L. pneumophila* Lens A) MG C) AG and polysaccharides produced by B) MG and D) AG *L. pneumophila* Lens. *Legionella* are in green and polysaccharides in blue. Pictures are representative of 6 independent experiments.

In order to determine whether the observed phenotypes for *L. pneumophila* in biofilm depended on the existence of a pre-formed biofilm, both types of *L. pneumophila*, MG and AG, were directly inoculated on the glass surfaces fixed at the bottom of the wells of a microplate. The MG *L. pneumophila* cells (figure 4A) appeared dispersed on the glass surface and this biofilm was found very thin, about 8 µm, with large areas devoid of attached cells. On the contrary, the biofilm obtained from the AG *L. pneumophila* (figure 4B) appeared thicker and presented densely packed bacteria separated by open structures.

Moreover, while the polysaccharides were poorly detected in the MG *L. pneumophila* biofilm (figure 4A) this type of macromolecule appeared abundant in the AG *L. pneumophila* biofilm (in blue on figure 4B and 4C) and was preferentially detected around the clusters of bacteria.

**Figure 5:**
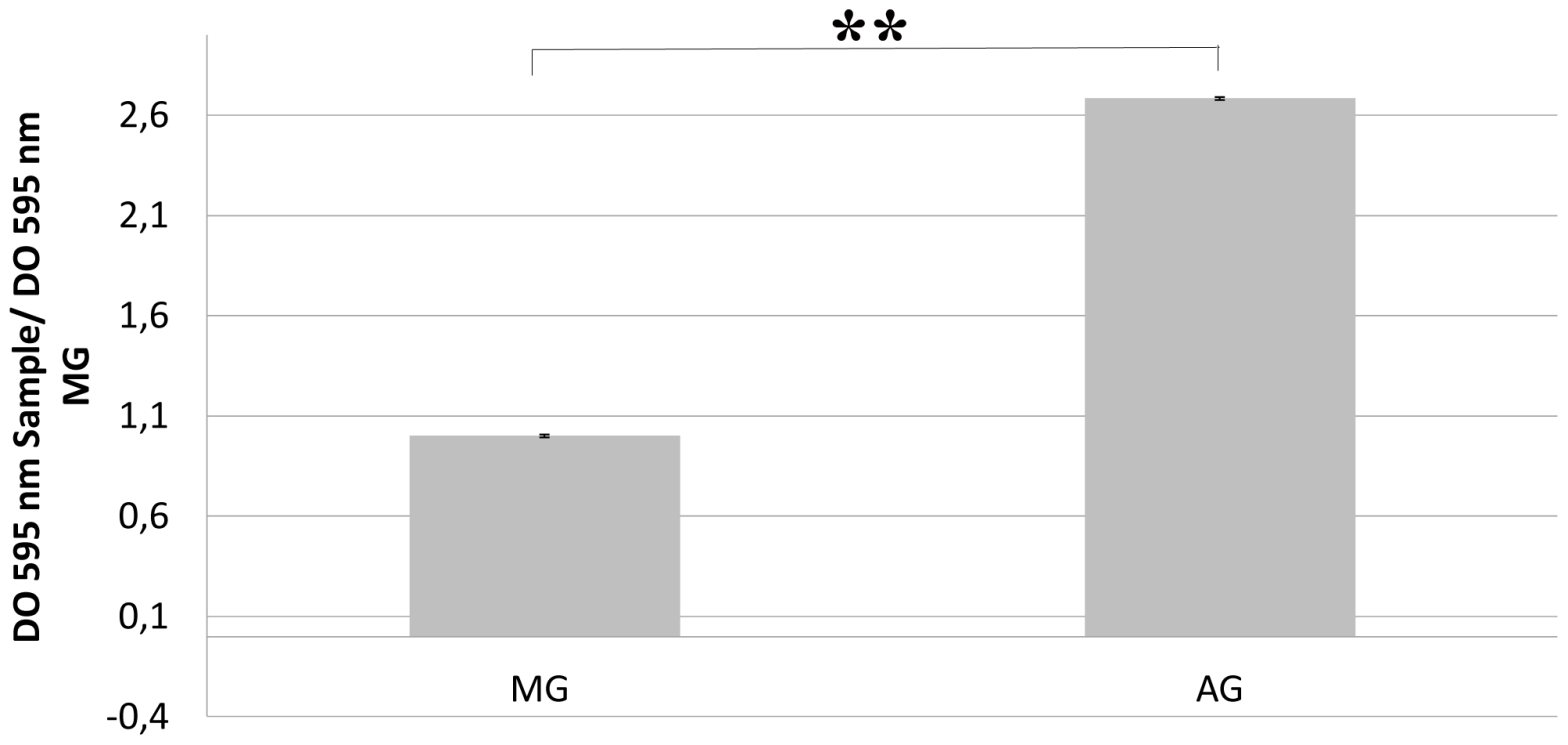
Alcian blue test results after 24^9^ MG and AG *L. pneumophila* Lens in Evian mineral water. Error bars indicate standard deviation from six independent experiments. The p-value is less than 0,0001, this difference is considered to be extremely statistically significant.

In order to verify whether the observed polysaccharides were newly synthesized by *L. pneumophila* and did not originate from *A. castelanii,* the amount of polysaccharide was determined using Alcian Blue. The amount of polysaccharide was too low to be detected in the MG as well the AG *L. pneumophila* suspensions used to seed the microplate wells (2.10^9^ cells), indicating that it was synthesized by bacteria during biofilm formation. Moreover, it was found that the amount of polysaccharide newly synthesized after one day was 2.78 times higher for the AG *L. pneumophila* than the MG *L. pneumophila* (Figure 5), which confirmed the microscopy analyses.

### *L. pneumophila* clusters origin

**Figure 6:**
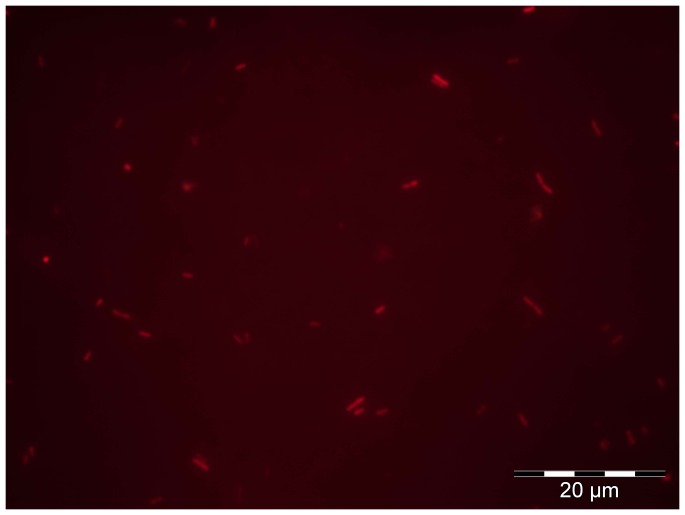
Epifluorescence microscopy observation of *L. pneumophila* Lens expressing ds-Red marker (in red) immediately after amoebae release. Picture is representative of 2 independent experiments.

We hypothesized that the bacterial clusters observed for AG *L. pneumophila* could be formed in the amoeba cell before the release of the bacteria in the medium or by aggregation only after the release of *L. pneumophila* in the very early step of the biofilm formation. The AG *L. pneumophila* suspension appeared only as isolated cells (figure 6). No bacterial clusters were observed in this experiment whatever the *L. pneumophila* strain, Lens (figure 4), Paris (not shown) or *L. longbeachae* (not shown). This indicates that the aggregation occurred after the release from amoeba.

**Figure 7:**
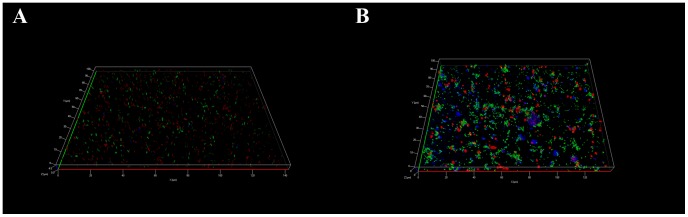
Apotome imaging after 7 days of biofilm development on a glass surface of a mixture of A) AG *L. pneumophila* Lens or B) MG *L. pneumophila* Lens expressing Ds-Red marker (in red) or GFP marker (in green) and polysaccharides (in blue). Pictures are representative of 3 independent experiments.

In order to verify whether the clusters originated from an aggregation of bacteria or the multiplication from a single cell, GFP and Ds-Red expressing AG *L. pneumophila* were mixed before spreading on the glass surface. The resulting clusters (Figure 7) were constituted of both types of cells, green and red, indicating that they came from an aggregation instead of cell division. Moreover, we observed a high quantity of yellow spots, corresponding to a co-localization of green and red cells. The same experiment, conducted with MG *L. pneumophila*, showed only isolated green and red cells homogenously distributed overall the glass surface (Figure 7).

**Figure 8:**
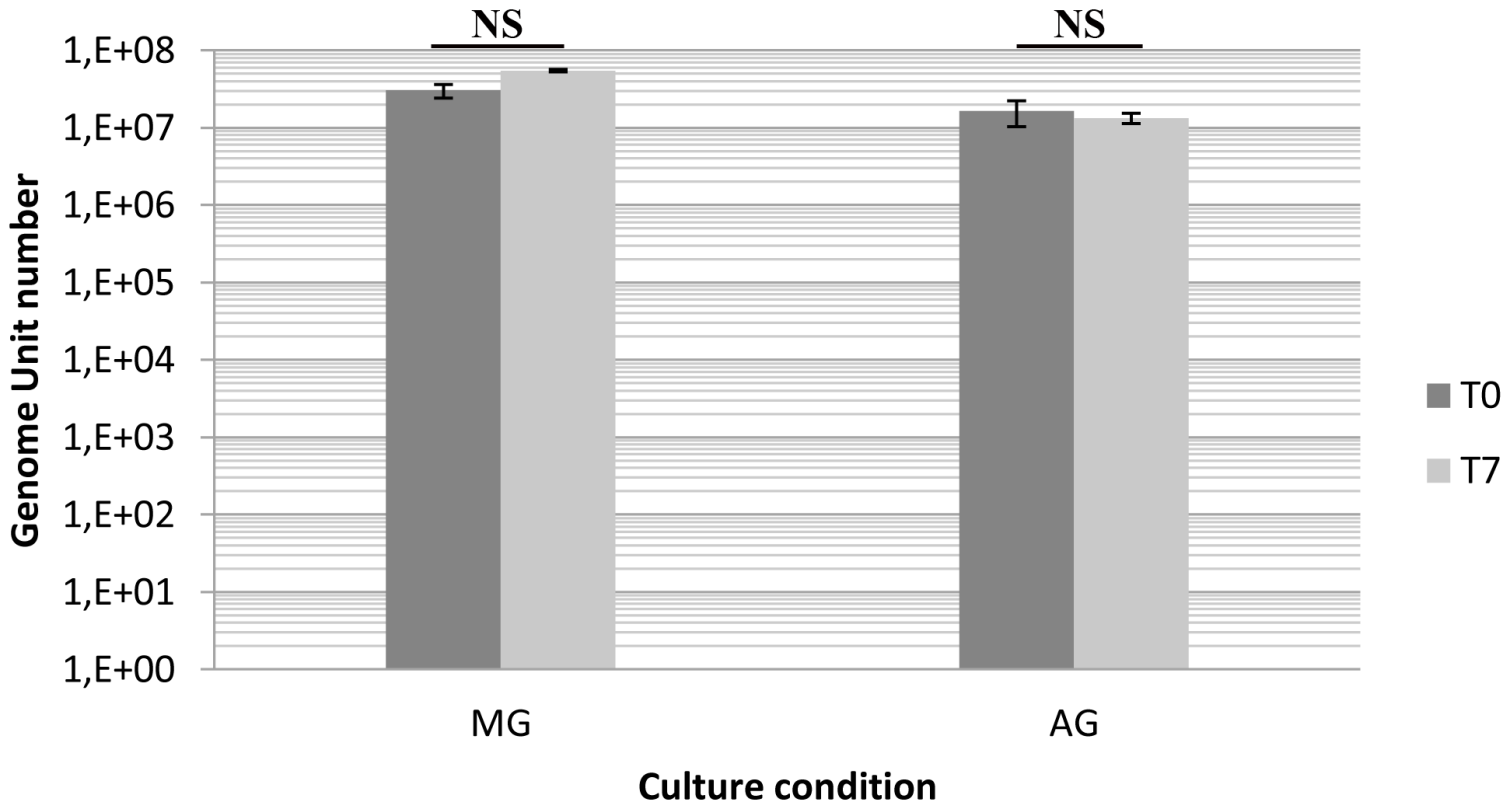
Results of qPCR amplification *mip* gene from MG and AG *L. pneumophila* Lens deposited on glass surface in Evian mineral water at T = 0 and T = 7 days. Error bars indicate standard deviation from three independent experiments. The p-value is equal to 0,5913 for AG *L. pneumophila* Lens and 0,2709 for MG *L. pneumophila* Lens. It is considered not to be statistically significant (NS).

Moreover, the non-multiplication of *L. pneumophila* was confirmed using a qPCR analysis since the number of MG a well as AG bacteria was similar, after one week biofilm development than in the initial suspension (figure 8). Declerck et al (2005) show that *L. pneumophila* increase in amoeba buffer, alone or with the same four non-*Legionella* strains, was only due to intracellular multiplication of the human pathogen [32].

Taken together, these results suggest that the *L. pneumophila*, which multiplied within the amoeba, expressed, after their release in the environment, a molecular factor, which induced a mutual attraction of the bacteria.

### Chemotaxis factor

**Figure 9:**
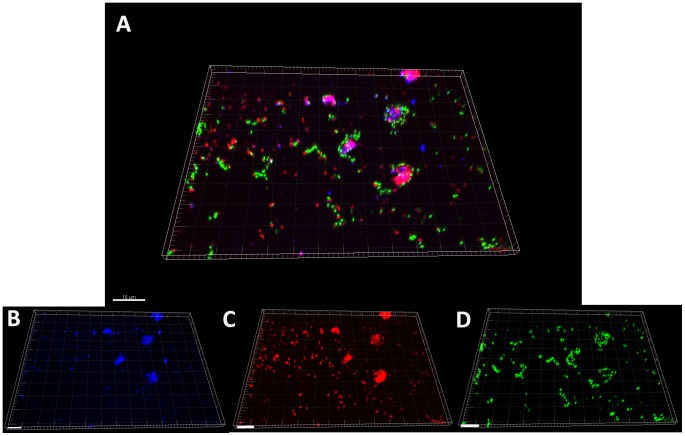
Apotome imaging after 7 days of biofilm development on a glass surface of a mixture of *L. pneumophila* lens AG expressing ds-Red marker (in red), and MG GFP marker (in green). Polysaccharides appear in blue. Pictures are representative of 3 independent experiments. Scale bar corresponds to 10 µm.

In order to verify our chemotaxis hypothesis, GFP expressing MG *L. pneumophila* were added to Ds-Red expressing AG *L. pneumophila* before inoculating the glass surface. The observed clusters of bacteria (figure 9) were composed of AG *L. pneumophila*, in red, covered with MG *L. pneumophila*, in green. This result shows that the “chemotaxis” factor expressed by AG bacteria was able to attract the MG *L. pneumophila*. Interestingly it has to be noticed that these “hybrid” clusters were partially embedded in newly synthesized polysaccharides (in blue, figure 9).

**Figure 10:**
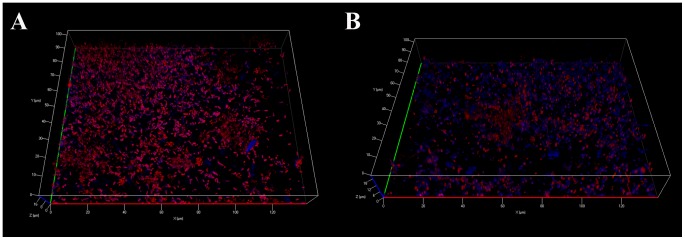
Apotome imaging after 7 days of biofilm development on a glass surface of a A) *L. pneumophila* Lens treated with supernatant of AG *L. pneumophila* Lens or B) with supernatant of AG *L. longbeachae*. Bacteria are in red and polysaccharides appear in blue. Pictures are representative of 3 independent experiments.

To confirm the factor production, MG *Legionella pneumophila* Lens were treated with the supernatant of AG Legionella then inoculated into plates. After 7 days of incubation, *Legionella* aggregates were visible (Figure 10) with the presence of a large amount of exo-polyssacccharides. These *Legionella* acquired the same ability to develop a biofilm as AG *Legionella*, which suggests the latter secreted a compound that can induce an AG phenotype. Moreover, this result confirms that the observed exopolysaccharides were produced by *Legionella pneumophila* and not by amoeba.

**Figure 11:**
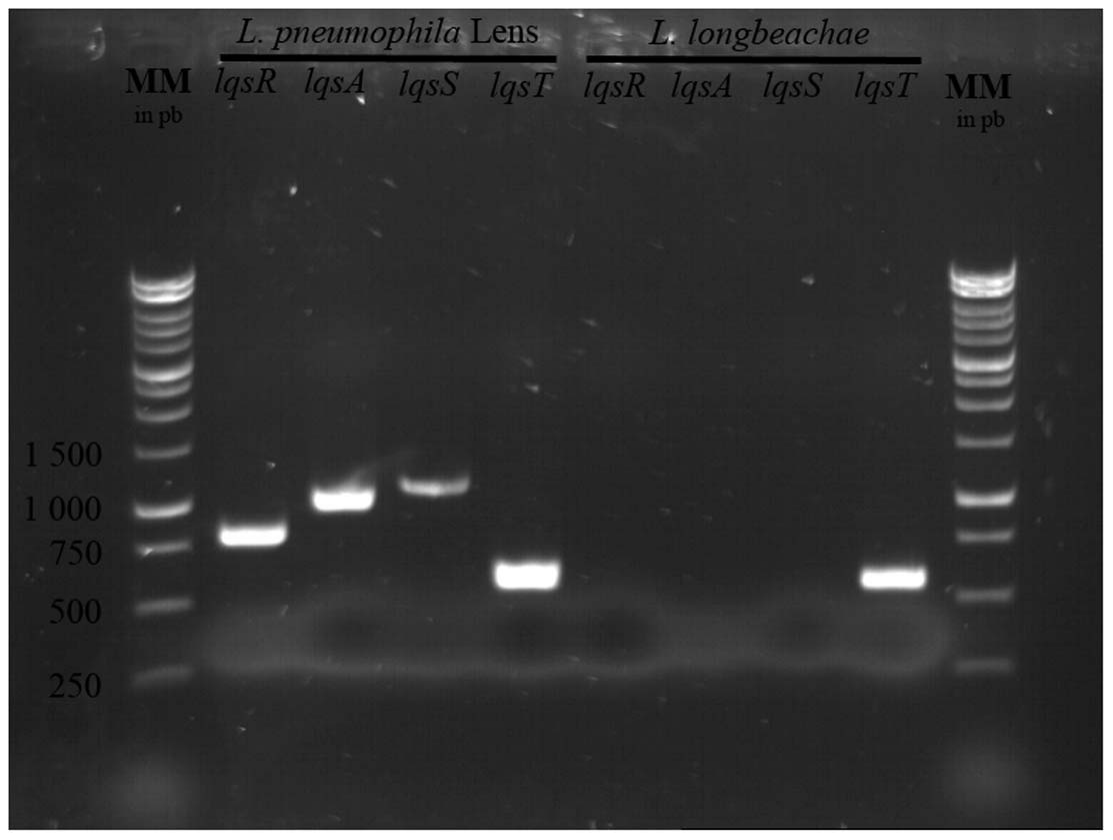
Picture of *lqs* PCR analysis electrophoresis gel of *L. pneumophila* Lens and L. *longbeachae*. Pictures are representative of 3 independent experiments.

These results suggest the implication of a system similar to quorum sensing. A *Legionella* quorum sensing system named *lqs* was described by Spirig et al [38]. It was shown that this system was absent from *Legionella longbeachae* [33], [39]–[42]. Firstly, a PCR analysis was performed to verify the presence of the different *lqs* genes in *Legionella pneumophila* Lens, used as a positive control and *Legionella longbeachae* (ATCC 33484) (Figure 11). The results of the amplification confirmed the absence of the *lqs*A, R and S from *Legionella longbeachae*. To determine whether the induction system detected here corresponds to *lqs* system, we performed an experiment in which MG *L. pneumophila* Lens were treated with the supernatant of AG *L. longbeachae*, then suspended in mineral water and deposited on glass plate. After 7 days of incubation, *Legionella* aggregates, similar to those obtained with *Legionella* AG, and presence of exo-polysaccharides were observed (Figure 10 B). Thus, despite the lack of *lqs* system in *L. longbeachae* including *lqsA*, coding the enzyme responsible for the synthesis of LAI-1 autoinducer, a biofilm phenotype was induced. These preliminary results suggest the existence of a different quorum sensing system in *Legionella* intra and inter-species.

## Discussion

It was demonstrated by numerous studies [13], [14], [18] that *L. pneumophila,* prepared from laboratory culture medium, is able to implement into a pre-formed biofilm for bacteria. In the first part of our study we showed that this implementation also occurred for *Legionella* cells released from ciliated protozoan or amoeba. And we also showed that the *Legionella* remained stable after seven days. This could indicate that the *Legionella* did not multiply in the biofilm, as it was previously proposed by Mampel and colleagues [33] who related the *L. pneumophila* development in biofilm to the adhesion of planktonic bacteria rather than by clonal replication of sessile cells. This is consistent with the study of the *Legionella* monospecies biofilms of Pecastaings and collaborators [43], who demonstrated that sessile *L. pneumophila* is able to grow in a minimum medium only when it is supplemented with iron and cysteine but not in an oligotrophic medium.

Whatever the origin of the bacteria, in our experiments, the *Legionella* cells appeared located on the surface of the pre-formed biofilm, which could be quite normal but also paradoxical, considering that the biofilm matrix protects the bacteria from external stresses. This superficial location could be related to our experimental conditions because observations were made only a week after seeding *Legionella*. It could be possible that the *Legionella* kinetic of penetration, or burying, into the biofilm matrix requires longer duration. However it could also be postulated that this superficial location on biofilm is advantageous regarding *Legionella*'s ability to grow on dead bacteria. Indeed, probably because of its requirement for L-cysteine, *Legionella* is capable of necrotrophic feeding, thus utilizing dead bacteria, protozoans or other microflora for sustenance [16]. In these cases, *Legionella pneumophila* is located on boundary layer of biofilm, an advantage for this fastidious organisms that permit to *Legionella* to have access at more nutrient [44]. Stress, like nutrient depletion as in our experiment, was described to promote acceptance and recruitment of new members in the biofilm [45], [46]. In another way, it could be proposed that the *Legionella* located at the surface of the biofilm in order to be more accessible for amoeba grazing then subsequent intra-amoeba multiplication.

The other major observation we made in this study is the presence of aggregates, conjugated with the production of exopolysaccharides by the “Amoeba Grown” (AG) *Legionella* cells but not the “Medium Grown” (MG). Moreover, this behavior is not related to the origin of the strain because the results obtained with a clinical strain, *L. pneumophila* Lens, and strain originally isolated in the environment, *L. pneumophila* Paris, as well as *L. longbeachae* were the same.

At first we hypothesized that these bacterial aggregates corresponded to micro-colonies obtained consecutively to clonal multiplication of *L. pneumophila* after the initial adhesion on the glass surface or the pre-formed biofilm. We demonstrated that aggregation depends on the mutual attraction of the bacteria but only after they grew within amoeba. Moreover, this is clearly in relation with a multiplication phase, since after passage in *Tetrahymena tropicalis*, where no replication was observed, a phenotype similar to the MG *Legionella*, not AG, was obtained. It can be hypothesized that this gathering is related to the production of a chemotaxis molecular agent specifically by the *Legionella* cells which grew in amoeba and after their release in the environment.

The target of this attraction could be bacterial cells themselves which could express both the chemotaxis factor and its putative specific sensor, the latter being induced by the factor acting as an inducer. In this case, we could postulate that the expression of the sensor was already induced for AG bacteria, which can rapidly gather whereas the MG *Legionella* expressed the sensor with delay when induced by the chemotaxis factor. This could then explain why the MG bacteria appeared to coat the surface of the AG clusters whereas we could imagine that the attraction of the MG bacteria to AG cells led to the formation of bi-colored aggregates, because both types of cells where mixed before deposition on glass surface. Moreover, the chemotataxis factor seems to be dependent of the intracellular replication as shown by its lack from CG *L. pneumophila*.

A *L. pneumophila* quorum sensing system involving an autoinducer, LqsA,and a sensor, LqsS, was previously described [40]. However, the authors have demonstrated that the genes encoding such system are not present in the *L. longbeachae* genome [38]. Nevertheless, the ability to form biofilm acquired by *Legionella* after its intracellular growth was observed in our experiments with MG *L. pneumophila* Lens treated with supernatant of AG *Legionella longbeachae* which did not possess *lqs* genes. Thus, the chemotaxis factor is different than LAI-1. Nevertheless, with this study, we have demonstrated the existence of new quorum sensing system intra and inter-species.

Finally, it has to be noticed that the AG *L. pneumophila* expressed, when adhered, large amounts of polysaccharides. Moreover, because AG cells but not MG cells produced very important amounts of such polysaccharides, this is again related to a passage within amoeba. In our opinion this phenomenon is developed by *Legionella* issued from the protozoan in order to rapidly protect themselves from external conditions. Indeed, when we first tried to quantify the *L. pneumophila* using classical crystal violet staining (not shown), these assays always gave negative results whereas qPCR as well as microscopy demonstrated the presence of a biofilm. We thus hypothesized that the polysaccharides in which the *Legionella* were embedded could trap the crystal violet which was not able to stain the bacterial cells. Structure of the exo-polysaccharide has to be characterized. However, because it was recognized by concanavalin A [47], [48] and Alcian Blue [49], [50] it could be hypothesized that it is partly constituted of glucose and/or mannose residues.

To confirm the major importance of the *Legionella* exo-polysaccharide in the biofilm development it could be proposed to characterize it and after to search genes encoding potential glycosyl transferases which are involved in the production of such polymers by screening the genome of *L. pneumophila*. Moreover, it is important to isolate and characterize the causative agent of chemotaxis to study its role in the different stages of biofilm development and its hypothetical roles in intracellular replication stages or in virulence capacity.

To conclude, we have developed a biofilm model near to the water distribution system using Amoeba Grown *Legionella* which have higher ability to colonize or to develop biofilm in particular via exopolysaccharide secretion. We propose to encourage the use of this model to test or develop disinfection process rather than conventional models using *Legionella* derived from culture medium.
